# Bioactive Ibuprofen-Loaded PLGA Coatings for Multifunctional Surface Modification of Medical Devices

**DOI:** 10.3390/polym13091413

**Published:** 2021-04-27

**Authors:** Oana Gherasim, Gianina Popescu-Pelin, Paula Florian, Madalina Icriverzi, Anca Roseanu, Valentina Mitran, Anisoara Cimpean, Gabriel Socol

**Affiliations:** 1 Lasers Department, National Institute for Lasers, Plasma and Radiation Physics, 409 Atomistilor Street, RO-077125 Magurele, Ilfov County, Romania; oana.gherasim@inflpr.ro (O.G.); gianina.popescu@inflpr.ro (G.P.-P.); 2 Department of Science and Engineering of Oxide Materials and Nanomaterials, Faculty of Applied Chemistry and Materials Science, Politehnica University of Bucharest, 1-7 Gheorghe Polizu Street, RO-011061 Bucharest, Romania; 3 Ligand-Receptor Interactions Department, Institute of Biochemistry, Romanian Academy, 296 Splaiul Independentei, RO-060031 Bucharest, Romania; florian_paula@yahoo.com (P.F.); radu_mada@yahoo.co.uk (M.I.); anca.roseanu@biochim.ro (A.R.); 4 Department of Biochemistry and Molecular Biology, Faculty of Biology, University of Bucharest, 91-95 Splaiul Independentei, RO-050095 Bucharest, Romania; valentina.mitran@bio.unibuc.ro (V.M.); anisoara.cimpean@bio.unibuc.ro (A.C.)

**Keywords:** biodegradable coating, long-term release, metallic implants, topical use, macrophages, fibroblasts, keratinocytes

## Abstract

To modulate the biofunctionality of implantable medical devices commonly used in clinical practice, their surface modification with bioactive polymeric coatings is an attractive and successful emerging strategy. Biodegradable coatings based on poly(lactic acid-*co*-glycolic acid), PLGA, represent versatile and safe candidates for surface modification of implantable biomaterials and devices, providing additional tunable ability for topical delivery of desired therapeutic agents. In the present study, Ibuprofen-loaded PLGA coatings (PLGA/IBUP) were obtained by using the dip-coating and drop-casting combined protocol. The composite materials demonstrated long-term drug release under biologically simulated dynamic conditions. Reversible swelling phenomena of polymeric coatings occurred in the first two weeks of testing, accompanied by the gradual matrix degradation and slow release of the therapeutic agent. Irreversible degradation of PLGA coatings occurred after one month, due to copolymer’s hydrolysis (evidenced by chemical and structural modifications). After 30 days of dynamic testing, the cumulative release of IBUP was ~250 µg/mL. Excellent cytocompatibility was revealed on human-derived macrophages, fibroblasts and keratinocytes. The results herein evidence the promising potential of PLGA/IBUP coatings to be used for surface modification of medical devices, such as metallic implants and wound dressings.

## 1. Introduction

The fabrication of bioactive coatings for clinically used biomedical materials and devices represents an attractive and challenging approach to refine interactions between host cells and biomaterials and to improve devices’ biofunctional performances [1,2]. To provide maximal therapeutic effects or to selectively tune final applications, implants, wound dressings, tissue engineering structures and auxiliary internal devices are suitable candidates for surface coating.

Polymeric biomaterials have an essential role in modern biomedical applications, not only for the development of new and effective drug delivery systems [3,4], specific and selective detection platforms [5,6] and multifunctional scaffolds for tissue engineering [7,8], but also for the fabrication of protective and bioactive coatings able to induce or modulate the biological response of commercially available products [9,10]. The particular use of polymeric coatings relies on their intrinsic features, including bioavailability and application-related tunable properties, versatile surface chemistry and solubility/dissolution, biodegradability and biocompatibility and genuine biological and therapeutic activity [11,12]. Moreover, by properly tuning their composition and microstructure, such coatings possess the ability to act as protective and bioactive matrices for therapeutic substances and to concurrently facilitate controlled and targeted local effects and diminish or eliminate collateral or side effects [13,14,15,16].

Dip-coating, a simple, reliable and reproducible technique, provides an inexpensive solution for the fabrication of protective thin or thick films with relatively precise control over layer thickness [17,18]. The method implies the immersion of a substrate, at a constant speed, into a solution that contains the material to be deposited. A homogeneous liquid film is obtained and the subsequent solvent evaporation at room temperature (RT) results in the formation of the desired coating. To obtain films with specific thickness and properties, solution composition and concentration, solvent features (nature, polarity, solubility and volatility), immersion time, withdrawal speed and environmental humidity can be modified during the dip-coating process [19,20].

The dip-coating technique proved an efficient approach to overcome the limitations of conventional metallic implants, such as bioinertness, osseointegration and long-term structural and functional performances. Such coatings have been studied out of the desire to improve the bioactivity (by encouraging initial adhesion of host cells onto the implant and by supporting normal cellular growth) and corrosion resistance of metallic implants [21,22]. Moreover, successful results have been reported by using polymeric films for surface modification of materials used in wound healing applications, as they enable the fabrication of specific hydrophilic/hydrophobic devices, with enhanced stability and fluid absorption, controllable permeability and tunable swelling behavior [23,24]. In addition, they have been evaluated as particular biofunctional interfaces, which possess anti-fouling activity, antimicrobial efficiency, local drug release ability, immunomodulatory role and wound healing effects [25,26,27,28].

The surface modification of implants with biodegradable polymers, which serves as controlled and targeted drug delivery systems, is a promising area of the biomedical field [29,30]. The clinically approved poly(lactic acid-*co*-glycolic acid) (PLGA) can be easily processed, and its features (including physical properties, chemistry, mechanics and degradability) can be operated to support a specific need [31,32]. Following PLGA’s biodegradation, the resulted lactic acid (LA) and glycolic acid (GA) are eliminated through the body by normal metabolic processes [33,34]. Various formulations based on PLGA have been successfully evaluated for biomedical applications, as their characteristics and biological functions depend on the molecular weight and ratio of lactide to glycolide monomers [34,35]. Furthermore, the degradation degree of PLGA copolymers is in accordance with the LA/GA ratio (PLGA 50:50 > PLGA 65:35 > PLGA 75:25 > PLGA 85:15) [36,37]. As for PLGA’s physical properties, there are multiple factors on which they depend, such as LA/GA ratio, initial molecular weight, storage temperature and water exposure. Since the degradation rate can be tailored by adjusting the LA/GA ratio, it has been reported that the embedding of a drug in PLGA-based biodegradable coatings resulted in controlled delivery of the therapeutic agent and reduced postoperative complications [38,39].

Ibuprofen (IBUP), a non-steroidal anti-inflammatory drug (NSAID), also known as an analgesic and antipyretic agent, acts as a reversible inhibitor for cyclooxygenase (COX-1 and COX-2) enzymes through steric hindrance, thus reducing the level of prostaglandins (resulting in diminution of pain, fever and acute inflammatory reactions) [40,41]. IBUP can be used for the fabrication of unconventional and effective polymer–drug conjugate systems [42,43]. For example, *in situ* formed PLGA implants proved the ability to simultaneously release Chlorhexidine and Ibuprofen, while maintained suitable mechanical properties, being thus proposed for the local treatment of periodontitis [44,45]. Hybrid coatings of titania nanotube array/PLGA embedded with IBUP demonstrated prolonged drug release and osteogenic differentiation potential [46,47]. The sustained slow release of IBUP from porous and hydrogel microspheres of PLGA copolymers outlined their potential use in the treatment of osteoarticular conditions [48,49]. Various biodegradable polysaccharides [50,51,52,53,54] and polyesters [55,56,57,58,59] have been evaluated as promising loading and releasing matrices for IBUP, enabling the development of unconventional and effective therapeutic systems. 

In our study, IBUP-loaded PLGA coatings (PLGA/IBUP) were proposed for the surface modification of implantable biomaterials, and showed controlled and localized drug release potential and improved biocompatibility. Relevant compositional and structural data were collected after biologically simulated dynamic studies. The composite coatings showed high *in vitro* cytocompatibility, as evidenced by enhanced adhesion and proliferation of human-derived macrophages, dermal fibroblasts and keratinocytes, representing the major cell types involved in wound healing. Thus, the developed PLGA/IBUP materials showed promising potential to be used as surface coatings for wound healing applications.

## 2. Materials and Methods

### 2.1. Materials

Poly(d,l-lactide-*co*-glycolide) copolymer (PLGA with 65:35 LA:GA ratio), Ibuprofen (IBUP, United States Pharmacopeia testing specifications), chloroform, acetone, ethanol and all reagents used for simulated body fluid (SBF) synthesis were acquired from Sigma-Aldrich (Merck Group, Darmstadt, Germany). All chemicals were of analytical purity and used without additional purification.

Sigma-Aldrich (Saint Louis, MO, USA) was also the provider of most reagents used for cellular evaluation: Minimal Essential Medium (MEM), Keratinocyte Serum-Free Medium (KSFM), phorbol 12-myristate 13-acetate (PMA), bovine serum albumin (BSA), bovine pituitary extract (BPE), fetal bovine serum (FBS), lipopolysaccharide (LPS from *Escherichia coli 055:B5*), phosphate buffer saline (PBS), epidermal growth factor (EGF), paraformaldehyde (PFA), glutaraldehyde (GA), hexamethyldisilazane (HMDS), Triton X-100, Tween 20, L-glutamine, sodium hydroxide, methanol and DAPI (6-diamidino-2-phenylindole) stain.

RPMI 1640 culture medium and streptomycin/penicillin (S/P) antibiotic mixtures were acquired from Gibco (Thermo Fisher Scientific, Waltham, MA, USA). CellTiter 96^®^ AQueous One Solution Cell Proliferation Assay (MTS) and Cell Counting Kit-8 (CCK-8) were purchased from Promega (Madison, WI, USA) and Sigma-Aldrich/Merck (Saint Louis, MO, USA), respectively. LIVE/DEAD^®^ Viability/Cytotoxicity Kit, Alexa Fluor^®^ 488 Phalloidin, Alexa Fluor^®^ 546 Phalloidin, Alexa Fluor^®^ 488 conjugated with anti-mouse anti-vinculin antibody (IgG) and ProLong™ Gold Antifade with DAPI blue dye were purchased from Invitrogen (Thermo Fisher Scientific, Carlsbad, CA, USA). Bio-Optica (Milano, Italy) provided the Sirius Red staining reagent, while Santa Cruz Biotechnology (Dallas, TX, USA) provided the mouse-derived anti-vinculin monoclonal antibody (IgG).

Human THP-1 monocytic cells (ATCC^®^ TIB-202™), CCD-1070SK fibroblasts (ATCC^®^ CRL-2091™) and KERTr keratinocytes (ATCC^®^ CRL-2309™) were provided by American Type Culture Collection (ATCC, Manassas, VA, USA). Medical grade 4 titanium (Ti) discs (12 mm diameter and 0.1 mm thickness) were acquired from a local supplier.

### 2.2. Synthesis of PLGA/IBUP Coatings

PLGA/IBUP coatings were obtained by combining two synthesis methods: dip-coating and drop-casting. Firstly, IBUP was dissolved in chloroform by sonication (1 min). Further, PLGA was added in the previous solution to obtain a 2:1 mass ratio between PLGA and IBUP, followed by 5 min of sonication. The as-resulted solution was used to obtain PLGA/IBUP (2:1 wt.%) coatings. Following the immersion of each Ti disc (10 s) and subsequent solvent evaporation, a volume of 60 µL from the same solution was pipetted on each sample’s side. For complete evaporation of chloroform, PLGA/IBUP-coated Ti samples were placed for 4 h in a laminar flow cabinet.

Before and after the coating procedure, Ti discs were weighed using a Mya 0,8/3.4Y analytical microbalance from Radwag (Radom, Poland). The mass of each PLGA/IBUP coating was determined as the difference between samples after and before the coating process, respectively. The obtained values (average values of five individual measurements) were considered for the quantitative evaluation of composite coatings under biologically simulated dynamic conditions.

For biological assays, PLGA, PLGA/IBUP (2:1) and PLGA/IBUP (10:1) coatings were obtained by using only the dip-coating protocol (immersion time of 10 s).

### 2.3. Calibration Curve of Ibuprofen

To obtain quantitative data on the release of IBUP from composite coatings during dynamic studies, the calibration curve of Ibuprofen was mandatory. Thus, we prepared an initial stock solution of 1 mg/mL IBUP in SBF (pH = 7.4). Complete dissolution of the drug was possible after sonication and vortexing, using in this respect the Elmasonic X-tra 30H ultrasonic bath from Elma Schmidbauer GmbH (Singen, Germany) and the Vortex Mixer from Velp Scientifica (Usmate, Italy), respectively. Starting from the stock solution, dilutions were made to obtain solutions with 0.75, 0.5, 0.25, 0.125 and 0.0625 mg/mL IBUP concentrations. All solutions were analyzed using the Evolution 220 UV-Vis spectrophotometer equipped with Insight software (Thermo Scientific, Schwerte, Germany). Measurements were performed in the absorbance mode, in the 200–400 nm wavelength range.

### 2.4. Dynamic Study of Drug Release and Coating Degradation

To evaluate the biologically simulated behavior of PLGA/IBUP coatings, dynamic tests were performed using a manufactured multichannel cell connected to a high precision peristaltic pump from Ismatec (Cole-Parmer, Wertheim, Germany) and coupled to a TC120 heated circulating bath from Grant Instruments (Fisher Scientific, Vantaa, Finland). Briefly, two samples with uniform coatings’ mass were immersed in each channel containing 4 mL of SBF. The temperature of SBF was maintained constant (37 °C) for all testing periods and the flow rate was set to 1 mL/min.

After dynamic testing of PLGA/IBUP coatings (2, 4, 6, 10, 15, 20 and 30 days), samples were removed, rinsed with deionized water, dried in the laminar flow cabinet and weighed using the Radwag Mya 0,8/3.3Y microbalance. The as-obtained final masses (average values of five individual measurements), corresponding to degraded coatings, were used to estimate the average mass loss (Δm) of PLGA/IBUP materials during dynamic testing. The value of Δm was calculated for each testing time as the difference between initial and final mass of PLGA/IBUP coatings (corresponding to before and after dynamic studies, respectively) and provided substantial information on the degradation of composite coatings. In addition, the immersion medium from each channel was collected for UV-Vis spectrophotometric analysis, performed using the Evolution 220 UV-Vis equipment. Thus, relevant data on the IBUP release profile were obtained.

To evaluate the effects of biologically simulated dynamic testing on PLGA/IBUP coatings, the collected samples were complementary investigated in terms of composition and microstructure. Fourier Transform Infrared (FT-IR) Spectroscopy and Scanning Electron Microscopy (SEM) studies were respectively performed. The FT-IR data were collected in the attenuated total reflection (ATR) mode with an 8400S Shimadzu equipment (Shimadzu Europa GmbH, Duisburg, Germany). The spectral collection was recorded with 4 cm^−1^ resolution within the 4000–550 cm^−1^ wavenumber range, 50 individual scans being acquired per sample. SEM investigation was performed with a FEI Inspect S scanning electron microscope (Thermo Fisher Scientific, Hillsboro, OR, USA), at an acceleration voltage of 12.5kV. To reduce electric charges’ accumulation, all samples were capped with a thin gold layer.

### 2.5. Biological Evaluation of PLGA/IBUP Coatings

In order to determine the *in vitro* biological effects of proposed coatings on macrophages, fibroblasts and keratinocytes, metallic substrates were modified with PLGA, PLGA/IBUP (2:1) and PLGA/IBUP (10:1) coatings, using the dip-coating protocol. Before testing, all samples were sterilized by UV exposure for 3 h.

#### 2.5.1. Effects of PLGA/IBUP Coatings on Human Macrophages

Human-derived THP-1 monocytic cells were cultivated in L-glutamine-containing RPMI 1640 medium, supplemented with 10% (*v*/*v*) inactivated FBS and 1% (*v*/*v*) S/P antibiotic mixture, followed by incubation in standard conditions (37 °C, humid atmosphere, 5% CO_2_). Cultures were maintained at 2 × 10^5^ cells/mL cellular densities and monocytes resulted from passages 4–6 (cellular viability above 95%) were differentiated to mature macrophages after incubation with 100 ng/mL of PMA for 72 h. The resulted THP-1-derived macrophages were considered for further experiments.

CellTiter 96^®^ AQueous One Solution Cell Proliferation Assay provides a quantitative evaluation of viable cells, based on the dehydrogenase-mediated reduction of MTS (3-(4,5-dimethylthiazol-2-yl)-5-(3-carboxymethoxyphenyl)-2-(4-sulfophenyl)-2H-tetrazolium) dye to insoluble formazan crystals. This process only occurs in metabolically active cells and the amount of released formazan is proportional to the number of viable cells. Macrophage-differentiated THP-1 cells were seeded onto the metallic substrates, which were previously individually placed in 24-well plates (Nunc, Roskilde, Denmark) containing RPMI 1640 supplemented with 10% inactivated FBS and 1% S/P. After standard incubation for 24 h and 72 h, the culture medium was removed and replaced with 300 μL of pre-warmed fresh culture medium and 60 μL of MTS. After 15 min of incubation in the dark, a volume of 100 μL from supernatants was transferred to a 96-well plate for optical density (OD) measurement at 450 nm using a Mithras Berthold LB940 microplate reader (Berthold Technologies, Bad Wildbad, Germany). Cell viability was expressed as percentage relative to the control specimens (bare Ti).

To evaluate the qualitative effects of PLGA/IBUP coatings on THP-1 differentiated macrophages, fluorescence microscopy studies were performed in order to follow the distribution of actin filaments. After 3 days of standard incubation, cells were fixed with 4% PFA (15 min), permeabilized with 0.2% Triton X-100, blocked with 0.5% BSA-PBS mixture (1 h) and washed with PBS. Furthermore, the actin filaments were stained with red-labeled Alexa Fluor^®^ 488 Phalloidin in 0.5% BSA-PBS solution (1 h, RT) and nuclei with blue-fluorescent DAPI (4′,6′-diamidino-2-phenylindole dihydrochloride) dye (1 min, RT). Subsequently, the samples were washed with PBS and mounted on microscope slides with ProLong™ Gold Antifade agent, to provide prolonged fluorescence signals. The as-treated samples were examined using the ApoTome.2 cursor mode of a Zeiss Axiocam ERc5s Apotom microscope with AxioVision4.8 software (Zeiss, Oberkochen, Germany).

For SEM evaluation, THP-1 differentiated macrophages cultured on the surface of tested materials were washed with PBS and fixed for 20 min with 2.5% GA solution. Subsequently, the samples were subjected to dehydration with 70%, 90% and 100% ethanol solutions (twice for 15 min for each concentration) and further processed by two immersions of 3 min each in 50%, 75% and 100% HDMS-ethanol solutions. HDMS evaporation was carried out in an AURA 2000 M.A.C. cabinet (Euroclone S.p.A., Pero, Italy). An Apreo S electron microscope from FEI (Thermo Fischer Scientific, Waltham, MA, USA) was used to examine cells’ morphology.

The level of human tumor necrosis factor (TNF-α) pro-inflammatory cytokine released by the cells incubated with the supernatants from materials maintained for 3 and 6 days in RPMI 1640 medium without L-glutamine, supplemented with 5% FBS and 1% P/S, was measured by the sandwich enzyme-linked immunosorbent assay (ELISA, R&D Systems, Minneapolis, MN, USA). Cells stimulated with 50 ng/mL LPS (18 h) were considered the positive control. Briefly, 96-well plates (MaxiSorp, Nunc) were coated with anti-TNF-α specific monoclonal antibodies (24 h, RT). After repeated washing with 0.05% Tween-PBS and blocking with 1% BSA-PBS, cell supernatants were incubated for 2 h at RT and followed by a 2 h incubation with biotin-coupled human TNF-α detection antibodies. The streptavidin-HRP conjugate and the H_2_O_2_:TMB enzyme substrates (BD Biosciences, San Jose, CA, USA) were used to measure the cytokine level in the cell environment. The enzyme reaction was stopped with H_2_SO_4_ 2N and the OD was measured at 450 nm with the Mithras LB940 DLReady spectrophotometer (Berthold Technologies). The cytokine concentration was expressed in pg/mL based on a specific standard curve.

#### 2.5.2. Effects of PLGA/IBUP Coatings on Human Fibroblasts

Direct contact assays were performed to evaluate the effects of designed composite coatings on the viability, proliferation and morphology of human CCD-1070SK fibroblasts. The cells were seeded at an initial density of 10,000 cells/cm^2^ on each sample that was previously placed in individual wells of 24-well plates (Nunc) containing MEM supplemented with 10% FBS and 1% S/P mixture. Afterward, the cell-populated samples were subjected to standard incubation for 1, 3 and 6 days.

The cellular viability was qualitatively determined by fluorescence microscopy using the LIVE/DEAD^®^ Viability/Cytotoxicity Kit, as described elsewhere [60]. Briefly, the fibroblasts in contact with the analyzed substrates were washed with PBS, then stained with calcein acetoxymethyl (2 µM) and ethidium bromide (4 µM) for 10 min and followed by microscopic investigation using the Olympus IX71 microscope with Cell F imaging software (Olympus, Tokyo, Japan). The viable cells show a green fluorescence following the reaction of intracellular esterases with calcein acetoxymethyl, whereas the non-viable ones are red-labeled due to the diffusion of ethidium homodimer across the damaged cell membranes and binding to the nucleic acids.

Quantitative cellular proliferation was evaluated by performing the CCK-8 assay which consists of the reduction of WST-8 (2-(2-methoxy-4-nitrophenyl)-3-(4-nitrophenyl)-5-(2,4-disulfophenyl)-2H-tetrazolium monosodium salt) to water-soluble yellow formazan crystals by dehydrogenases from viable cells [61]. For this purpose, the cells were washed with PBS and subsequently incubated for 2 h in culture medium containing 10% *v*/*v* CCK-8 solution. Then, the OD of the reaction products was recorded at 450 nm using an Appliskan multi-well microplate reader (Thermo Scientific, Vantaa, Finland).

The effects of PLGA/IBUP coatings on CCD-1070SK fibroblasts were complementary evaluated by fluorescence microscopy and SEM studies of the cellular morphology. For fluorescent staining, the cells were fixed with 4% PFA, permeabilized and blocked with 0.1% Triton X-100/1% BSA for 1 h and then sequentially stained with Alexa Fluor^®^ 488 Phalloidin and DAPI [61]. Relevant micrographs were obtained using the Olympus IX71 inverted fluorescent microscope and Cell F image software. In addition, the fibroblast morphology was evaluated by SEM investigation at 24 h and 72 h post-seeding, as presented above.

#### 2.5.3. Effects of PLGA/IBUP Coatings on Human Keratinocytes

KSFM supplemented with BPE and EGF were added in each well of 24-well plates (Nunc), after the metallic samples were correspondingly placed. Human keratinocytes were seeded at a cellular density of 10,000 cells/cm^2^, followed by standard incubation (37 °C, humid atmosphere containing 5% CO_2_). Culture medium was replaced every three days.

The adhesion and morphology of KERTr cells grown on PLGA-based coatings were evaluated at 2 h and 24 h post-seeding, both by fluorescence microscopy and SEM investigation. To visualize keratinocytes in fluorescence microscopy, the cell-populated samples were sequentially incubated with primary mouse anti-vinculin monoclonal antibody, secondary anti-mouse IgG antibody conjugated to Alexa Fluor^®^ 488, and phalloidin-conjugated Alexa Fluor^®^ 546 followed by staining with DAPI, as previously reported [62]. Following the same protocol as in the case of fibroblasts, KERTr cells maintained in contact with the analyzed samples for 2 h and 24 h were processed for SEM investigation, which was performed using the Apreo S equipment.

Cellular viability and proliferation of human keratinocytes were investigated after 1, 3 and 6 days of standard incubation by complementary LIVE/DEAD^®^ and CCK-8 assays, respectively. Similar experimental conditions as in the case of the CCD-1070SK cell line were used.

#### 2.5.4. Statistical Analysis

For the biological studies, data were collected from triplicate samples and the results were expressed as mean values ± standard deviation (SD). Statistical analysis of the data was performed with the GraphPrism software (Version 4, San Diego, CA, USA) using one-way ANOVA or two-way ANOVA followed by Bonferroni’s multiple comparison test, used to test the statistical significance of results between the investigated groups. Differences with P values lower than 0.05 were considered statistically significant.

## 3. Results and Discussions

### 3.1. Calibration Curve of Ibuprofen

**Figure 1:**
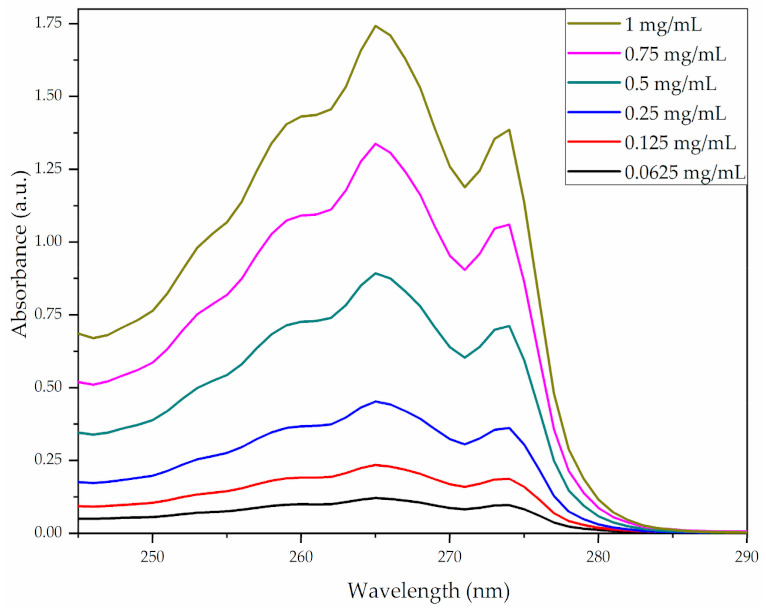
UV-Vis spectrograms of Ibuprofen solutions.

The measured absorbance of IBUP solutions evidenced specific concentration-dependent absorption bands at 265 and 274 nm wavelengths (Figure 1).

**Figure 2:**
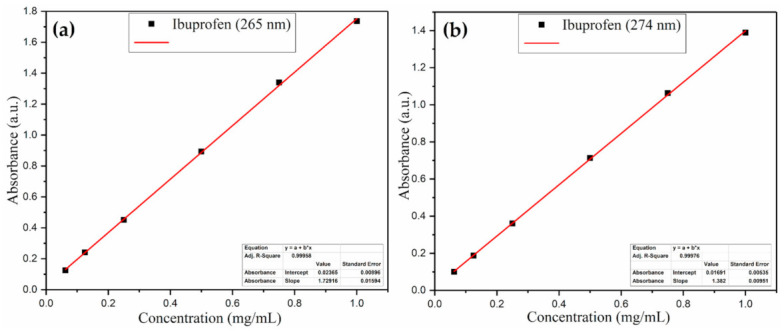
Standard calibration curve of Ibuprofen at 265 nm (**a**) and 274 nm (**b**).

Based on these spectrograms, precise absorbance values were identified and plotted against concentration, to obtain six-point standard calibration curves of Ibuprofen (Figure 2). The linear dependence between the measured absorbance and IBUP concentration was in agreement with the Lambert–Beer law, regardless of the monitored absorption band.

### 3.2. Dynamic Study of Drug Release and Coating Degradation

Time-dependent softening and degradation of resorbable coatings generally occur after their immersion in biological fluids, mainly due to comparable glass transition temperatures (Tg) of such materials with host organism’ thermal values, which result in harmless degradation of products that are susceptible to physiological metabolization [63,64]. Furthermore, if a drug is embedded within the crystalline lattice of a degradable polymeric coating, its intrinsic architecture enables the release of the active substance after specific events occurred under physiologically simulated conditions (solubilization, swelling, dissolution, erosion) [65,66,67].

**Figure 3:**
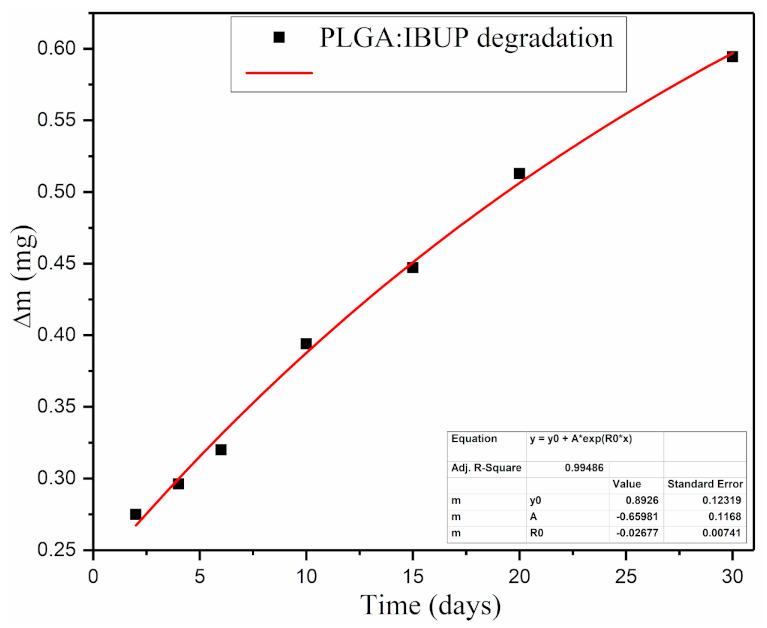
Degradation of PLGA/IBUP coatings during biologically simulated dynamic testing, evidenced by mass loss variation.

The combined dip-coating and drop-casting protocol enabled the obtaining of comparable PLGA/IBUP coatings, with a calculated average mass of ~3.676 mg. The degradation of composite coatings was evaluated based on the average mass losses (Δ*m*) of PLGA/IBUP coatings recorded for up to 30 days of testing under biologically simulated dynamic conditions. In fact, Δm directly provided the average mass of degraded materials. As evidenced in Figure 3, a progressive increase of Δm was recorded during dynamic studies, along with a slow degradation rate of the polymer matrix. A reduced mass loss was observed until day 6 of evaluation, whereas longer testing times (10, 15, 20 and 30 days) determined a significant increase in the coating’s degradation. The relative mass loss (%) of PLGA/IBUP coatings varied between ~7.89% and ~16.75%, corresponding to 2 and 30 days of dynamic studies, respectively.

**Figure 4:**
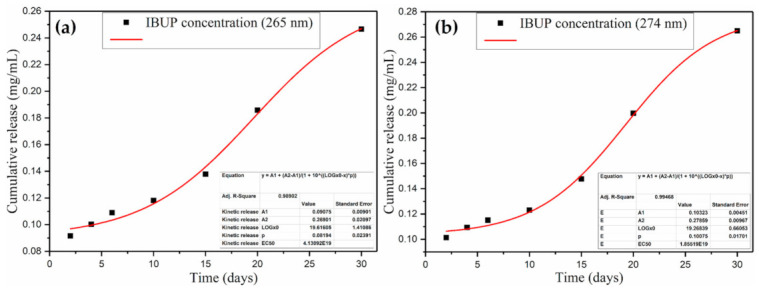
Cumulative release of Ibuprofen from PLGA/IBUP coatings during biologically simulated dynamic testing, obtained by monitoring the drug’s absorbance maxima at 265 nm (**a**) and 274 nm (**b**).

To determine the release profiles of Ibuprofen (Figure 4), absorbance variations of all solutions collected after dynamic testing were monitored at 265 and 275 nm and translated to the amount of Ibuprofen using the calibration curves. A reduced release of IBUP (maximum 20 µg/mL) was evidenced in the first 6 days of evaluation. Starting from day 10, a progressive increase in released drug was observed, accompanied by the increase in coating’s degradation (Figure 3). In compliance with previous results on PLGA/IBUP degradation, the concentration of IBUP released after longer time intervals (15, 20 and 30 days) recorded a significant and progressive increase. Drug release profiles were similar, regardless of the monitored wavelength. Based on the drug’s absorbance maxima from 265 nm, the concentration of released IBUP ranged between ~90 µg/mL (after 2 days) and ~250 µg/mL (after 30 days).

**Figure 5:**
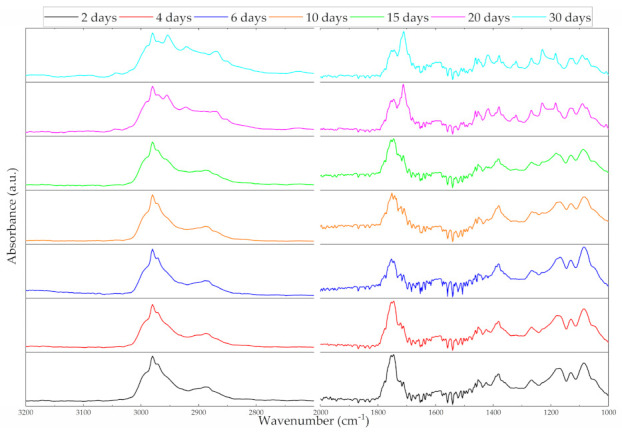
ATR-FTIR spectra of PLGA/IBUP coatings during biologically simulated dynamic testing.

All PLGA/IBUP coatings preserved their characteristic moieties following the performed dynamic studies, regardless of the testing time. This indicated that neither drastic alterations of materials occurred until the 30^th^ day, nor secondary products were formed. Specific IR maxima identified in Figure 5 were attributed to both PLGA and Ibuprofen as follows: asymmetric and symmetric stretching of –CH_3_ and –CH_2_ (between ~3000 and ~2850 cm^−1^), C=O symmetric stretching (between ~1760 and ~1690 cm^−1^), overlapped –OH bending and –CH_3_ symmetric deformation (~1385 cm^−1^) [68,69]. Vibrations corresponding to LA (methyl’s asymmetric deformation) and GA (methylene’s symmetric deformation) were identified at ~1450 cm^−1^, while characteristic vibrations of C–O–C moiety from both units were evidenced between ~1170 and ~1080 cm^−1^ [70,71]. With an increasing testing time, a diminish in the intensity of carbonyl originating from copolymer’s ester bonds (~1750 cm^−1^) was observed, accompanied by the appearance of a new peak at ~1710 cm^−1^. This lower wavenumber band was particularly associated with vibrations of hydrogen-bounded carbonyl from carboxylic acids, such as LA and GA resulted from SBF-mediated hydrolysis of PLGA [72,73]. At the same time, more pronounced maxima and spectral widening occurred in the 3000–2850 cm^−1^ region, as a result of overlapped –CH and O–H vibrations, also originating from carboxylic acids.

**Figure 6:**
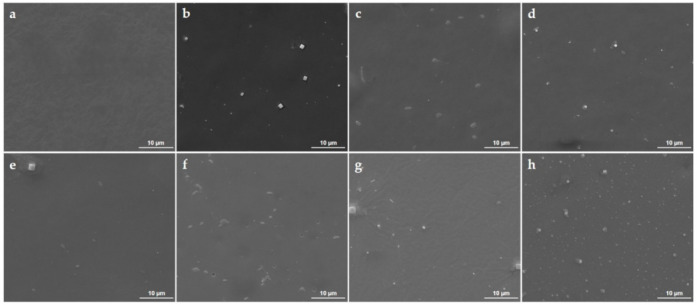
SEM images of initial PLGA/IBUP coating (**a**) and PLGA/IBUP coatings during biologically simulated dynamic testing: 2 (**b**), 4 (**c**), 6 (**d**), 10 (**e**), 15 (**f**), 20 (**g**) and 30 days (**h**).

Relevant microstructural aspects on the initial PLGA/IBUP coatings and their behavior after biologically simulated dynamic studies were provided by comparative SEM analysis (Figure 6). Uniform and continuous composite film was obtained by the dip-coating and drop-casting combined method (Figure 6a). At this level, the topography of metallic substrate could be noted (due to the formation of adherent coating), and no structural defects of initial PLGA/IBUP coating were evidenced. Time-dependent structural modifications and polyhedral aggregates (specifically assigned to the inorganic salts from SBF) were observed onto PLGA/IBUP coatings after dynamic testing.

After 2 days of evaluation, the textured appearance of PLGA/IBUP coating’s surface was evidenced and the presence of well-defined hill-like organic formations was noticed (Figure 6b). Such structural changes were attributed to the incipient swelling of copolymer after immersion in aqueous solution. Similar but reduced-in-size structural modifications were also observed after 4, 6 and 10 days of dynamic testing (Figure 6c–e). This behavior might indicate a substantial swelling of PLGA matrix after 2 days, followed by progressive swelling and local reorganization of polymeric chains [74,75], but also accompanied by incipient microstructural alterations (fine cracks were identified after the considered testing intervals). Microstructural modifications identified until the 10^th^ day of dynamic studies evidenced a slow and gradual alteration of PLGA/IBUP coatings, in compliance with previous results on mass loss and drug release.

From a structural point of view, prolonged testing times (15, 20 and 30 days) determined major structural changes, along with notable compositional modifications (Figure 5), significant mass losses (Figure 3) and increased drug release (Figure 4). After 15 days of evaluation (Figure 6f), numerous pits were noticed within the composite film due to the degradation of polymeric matrix. An additional 5 days of dynamic testing seemed to have a decisive role on the polymeric matrix, as interconnected cracks were evidenced at this time interval (20 days, Figure 6g). Due to the coating’s degradation, the substrate’s topography was evidenced. A pronounced degraded coating and more irregular and hydrophilic surface (due to local exposure of the substrate) were favorable for abundant deposition of salts originating from the testing medium. Moreover, once attached onto the already impaired coating, inorganic salts acted as local accelerators for polymeric degradation, by promoting hydrolysis of copolymer’s ester groups due to local pH changes [38,76]. These observations reinforced previous IR results on the SBF-mediated hydrolysis of PLGA matrix, evidenced by a significant shift of carbonyl moiety originating either from PLGA (until 15^th^ day of testing) or from LA and GA units (20 and 30 days of testing). Exfoliation was noticed in the vicinity of these inorganic aggregates, indicating the irreversible damage of PLGA/IBUP coating after 30 days of dynamic studies (Figure 6h).

If for testing intervals of 2, 4, 6, 10 and 15 days (Figure 6b–f), PLGA/IBUP coatings appeared to suffer rather reversible swelling phenomena (accompanied by the slow release of ~40 µg/mL of therapeutic agent), the longer testing times determined irreversible degradation (Figure 6g,h) and chemical modification (Figure 5) of the polymeric matrix (accompanied by the gradual release of ~120 µg/mL of Ibuprofen).

PLGA-based biomaterials possess intrinsic hydrophobicity, but their degradability in various inorganic or organic solvents can be experimentally tailored by properly adjusting the ratio between polymeric constituents. In addition, the variation of the LA:GA ratio directly impacts the copolymer’s crystallinity and *T*_g_ values. Thanks to their particular Tg variations (40–60 °C) [77,78], PLGA-based biomaterials are promising candidates for applications that require direct contact with structures of the human body. Because of the nontoxicity of PLGA and its degradation products, together with their non-immunogenicity and physiological metabolization, this copolymer is highly recommended for the fabrication of platforms that enable the controlled, targeted and triggerable delivery of therapeutic agents [79,80].

Our data evidenced the progressive long-term degradation of PLGA matrix and a cumulative release of IBUP of ~16.75% after one month. The results are in compliance with other studies on drug release from PLGA-based biomaterials under physiologically simulated conditions. PLGA nanofiber coatings provided a sustained release of aspirin for up to 2 months (after an early significant release in the first 2 weeks), while they improved the osseointegration and inhibited the peri-implant inflammation and osteolysis of as-modified Ti screws [81]. Sustained and prolonged release of bone morphogenetic protein-2 (BMP-2) was attained for dental implants modified with dip-coated BMP-2 layers entrapped within spray-coated PLGA layers. After 30 days, the cumulative release of protein varied between 17% and 23%, depending on the applied multilayers. The hydrophobic coatings promoted bone cells’ proliferation and stimulated mineralization, but also exhibited hemocompatibility and platelet activation ability [82]. Streptokinase-loaded PLGA membranes were successfully evaluated as coatings for nitinol stents, as they enabled a burst release of the antithrombotic agent after 3 days and its sustained slow release for 30 days. For a period of 60 days post-implantation, no allergic and immune reactions nor cardiac events were noticed [83].

At this point, we assert that the herein-proposed PLGA/IBUP coatings are promising materials for long-term surface modification of medical devices, as the biologically simulated dynamic studies evidenced the progressive degradation of copolymer matrix and the gradual release of Ibuprofen.

### 3.3. Biological Evaluation of PLGA/IBUP Coatings

#### 3.3.1. Effects of PLGA/IBUP Coatings on Human Macrophages

The surface characteristics of biomaterials influence macrophages’ activation status; they are key cells in orchestrating the immune response in endogenous regeneration process [84,85]. Their response to surface cues is important for the evaluation and improvement of material’s efficacy during the transition to *in vivo* studies. The dependence of cellular response on coatings’ characteristics was demonstrated in our previous studies performed on composites containing hydroxyapatite, Lactoferrin and polyethylene glycol-polycaprolactone copolymer [86].

**Figure 7:**
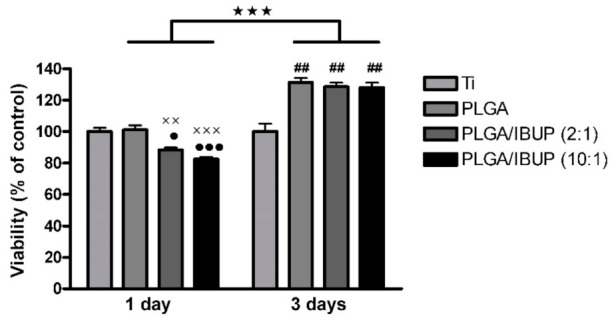
Viability of human macrophages grown in contact with PLGA-based coatings for 1 and 3 days (MTS assay); ^★★★^*p* < 0.001 vs. 1-day time point; ••• *p* < 0.001, • *p* < 0.05 vs. Ti; ^×××^ *p* < 0.001, ^××^ *p* < 0.01 vs. PLGA; ^##^ *p* < 0.01 vs. Ti.

In this work, THP-1 cells differentiated to macrophages were used to investigate the interaction with PLGA/IBUP coatings. Cell viability was quantitatively evaluated after 1 and 3 days of culture in direct contact with all surfaces by MTS colorimetric assay. As shown in Figure 7, all PLGA-based coatings induced a significant increase in cell viability (*p* < 0.001) at 3 days, compared to the 1-day time point. It can be observed that PLGA/IBUP (2:1) and PLGA/IBUP (10:1) coatings led to a decrease of THP-1 cell viability compared to both PLGA (*p* < 0.001 and *p* < 0.01) and Ti (*p* < 0.001 and *p* < 0.05) samples after 1 day of treatment. In contrast, increasing the time of cell-surfaces contact to 3 days resulted in the statistically significant increase of metabolically active cells grown on all PLGA coatings (*p* < 0.01 vs. Ti). It is worth mentioning that an increased release of IBUP from the analyzed coatings (according to drug release profiles from Figure 4) did not induce a negative effect on cell viability and metabolic activity. Moreover, our experiments performed with free Ibuprofen confirmed the absence of cytotoxic effects, with no less than 92% viability for concentrations up to 200 µg/mL (data not shown).

**Figure 8:**
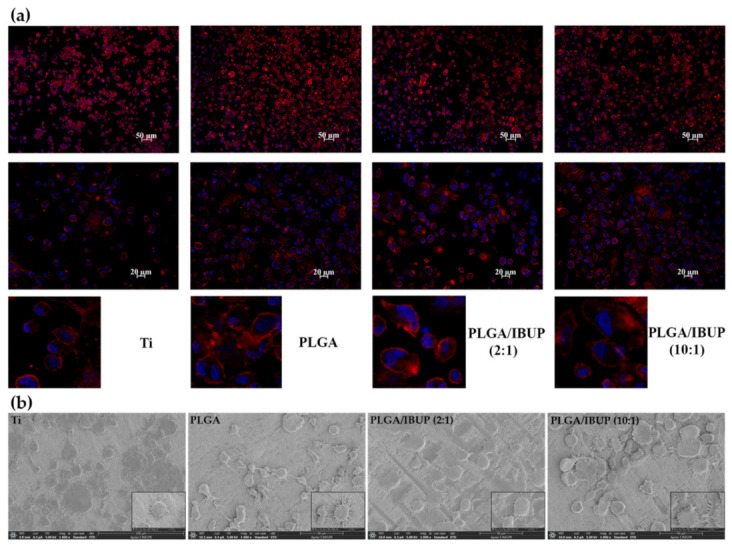
Morphological features of human macrophages on PLGA-based coatings after 3 days, evidenced by: (**a**) fluorescence microscopy showing actin filaments (red) and nuclei (blue), scale bar 50 and 20 µm; (**b**) SEM investigation, scale bar 50 µm.

The effects of the materials’ surface on cell adhesion, spreading and morphology were investigated by fluorescence microscopy and SEM. Fluorescence micrographs provided complementary data on the interaction of human macrophages with PLGA-based coatings (Figure 8a). A rounder morphology of THP-1 cells was evidenced in the case of control samples. In comparison, an increased adhesion of cells was noticed for metallic substrates modified with PLGA, PLGA/IBUP (2:1) and PLGA/IBUP (10:1) coatings, as evidenced by abundant fluorescent microfilaments within adherent cells. The THP-1 cells grown on Ti coated with PLGA/IBUP (both ratios) presented mixed spherical and elongated morphologies, due to intrinsic accommodation of cells to the characteristics of the surfaces.

Complementary data on the interactions between differentiated THP-1 cells and composite materials were provided by SEM micrographs (Figure 8b). Preferential round flatted morphology was evidenced for cells grown onto bare Ti substrate, while enhanced cellular spreading and abundant cellular extensions were noticed for all PLGA-coated specimens.

With regards to the inflammatory potential of the surface components, none of the coatings induced an inflammatory response, as evidenced by the ELISA assay (data not shown). The cells cultured in the presence of supernatants generated from PLGA-based materials did not release detectable levels of TNF-α, as compared with the 9435 ± 1164 pg/mL TNF-α value detected in the case of positive control (LPS-stimulated cells).

Altogether, these results evidenced improved cellular attachment and spreading, demonstrating the highly biocompatible behavior of PLGA-based materials with respect to THP-1 differentiated human macrophages. These findings are in agreement with our previous study reporting the biocompatibility of Ti coatings based on IBUP-encapsulated PLGA microspheres and their ability to promote the adhesion and proliferation of macrophages [87].

#### 3.3.2. Effects of PLGA/IBUP Coatings on Human Fibroblasts

Considering that fibroblasts and keratinocytes communicate with each other via double paracrine signaling loops, they are considered essential cells in the restoration of normal tissue homeostasis after the wounding process [88,89]. Hence, we further investigated their behavior on the newly developed PLGA-based coatings.

**Figure 9:**
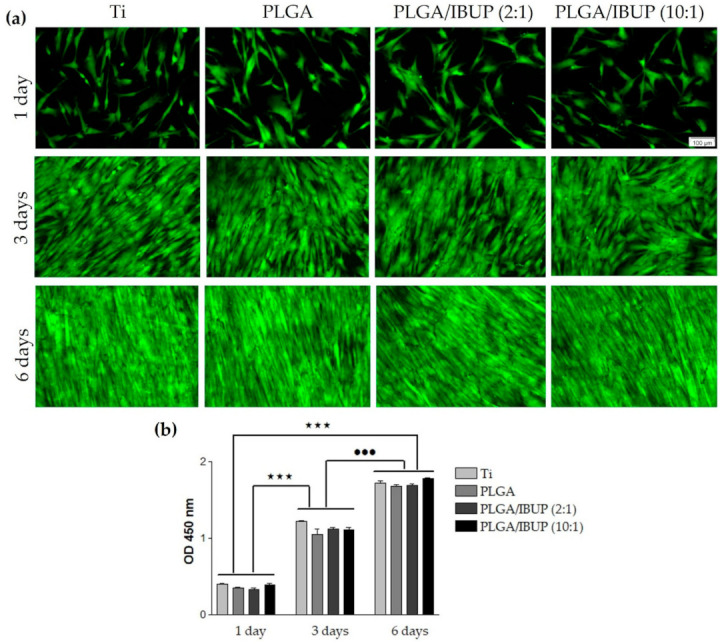
Viability and proliferation of human fibroblasts grown in contact with PLGA-based coatings for 1, 3 and 6 days, evaluated by: (**a**) viable cell fluorescent labeling (Live/Dead assay), scale bar 100 µm; (**b**) fibroblast proliferative potential (CCK-8 test); ^★★★^ *p* < 0.001; ^•••^ *p* < 0.001 vs. 3-day time point.

Thus, to evaluate the survival of CCD-1070SK fibroblasts in contact with bare Ti (control) and Ti substrates modified with PLGA, PLGA/IBUP (2:1) and PLGA/IBUP (10:1) coatings, qualitative Live/Dead test was performed at 1, 3 and 6 days post-seeding (Figure 9a). Regardless of the testing time, the collected fluorescence micrographs evidenced a time-dependent increase in the number of viable cells (green labeled), with comparable cellular densities between the samples. These results showed the ability of PLGA/IBUP coatings to sustain the cellular survival and promote the proliferation of fibroblasts throughout the experimental period, as no dead cells (red labeled) were evidenced. In compliance with these results, the quantitative CCK-8 assay (Figure 9b) revealed a time-dependent significant increase (*p* < 0.001) in the number of viable fibroblasts from day 1 to 3- and 6-day time points. Furthermore, no significant differences between control specimens (uncoated Ti) and samples modified with PLGA-based coatings were noticed, suggesting that these coatings are suitable candidates for promoting wound healing.

**Figure 10:**
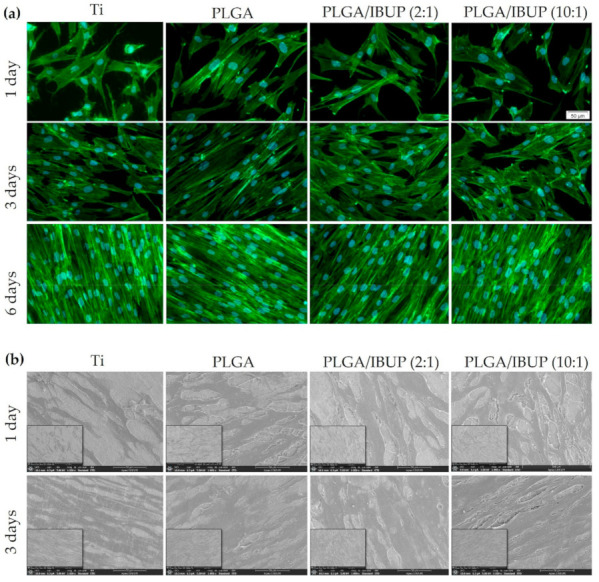
Morphological features of human fibroblasts on PLGA-based coatings, evidenced by: (**a**) fluorescence microscopy showing actin filaments (green) and nuclei (blue), scale bar 50 µm; (**b**) SEM investigation, scale bar 50 µm.

Relevant details on the adhesion and morphology of CCD-1070SK cells after their interaction with PLGA-based coatings were provided by fluorescence microscopy studies (Figure 10a). For all samples and all considered testing intervals, the fibroblasts were fully spread onto the surface of specimens, possessed processes extending from their cell bodies and presented typical elongated and spindle-shaped morphology with well-defined parallel actin filaments and prominent elliptical nuclei. The presence of abundant cytoplasmic extensions and filopodia, as essential indicators for the attachment and spreading of cells to/on specimens’ surface, was noticed starting from day 1 of evaluation. Cellular density increased with the incubation time, until confluency (reached after 6 days). 

The fluorescence microscopy results were reinforced by SEM data (Figure 10b), which demonstrated the beneficial effects of PLGA-based coatings on the attachment and spreading of CCD-1070SK cells, in comparison with bare Ti (control). After 1 day of incubation, flattened and elongated cells were distinguished, with more abundant cytoplasmic extensions when cultivated on Ti modified with PLGA and PLGA/IBUP coatings. No significant differences were observed in surface coverage after 3 days of culture. Altogether, the fluorescence microscopy and SEM investigation confirmed the highly cytocompatible feature of PLGA/IBUP coatings with respect to human fibroblasts.

**Figure 11:**
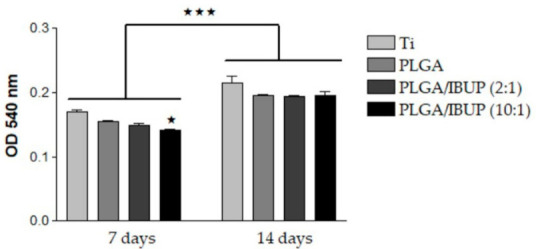
Collagen synthesis of human fibroblasts grown on PLGA-based coatings for 7 and 14 days (Sirius Red assay), ^★^ *p* < 0.05 vs. uncoated Ti substrate; ^★★★^ *p* < 0.001 vs. 7-day time point.

Fibroblasts are essential active players during the inflammatory, proliferative and maturation phases of normal wound healing, being involved in the formation of several extracellular matrix components, including collagen [90,91]. As PLGA/IBUP coatings were intended for the surface modification of common biomedical devices (including metallic implants and wound dressings), the ability of the proposed coatings to modulate the collagen synthesis ability of CCD-1070SK cells was necessary. As shown in Figure 11, PLGA-based materials determined a time-dependent increase in collagen synthesis by fibroblasts (*p* < 0.001). No significant differences between investigated samples were noticed for the same testing time, except for the PLGA/IBUP (10:1) coating, which recorded a significant decrease (*p* < 0.05) in collagen synthesis at the 7-day time point, but this difference disappeared after 14 days of culture.

#### 3.3.3. Effects of PLGA/IBUP Coatings on Human Keratinocytes

**Figure 12:**
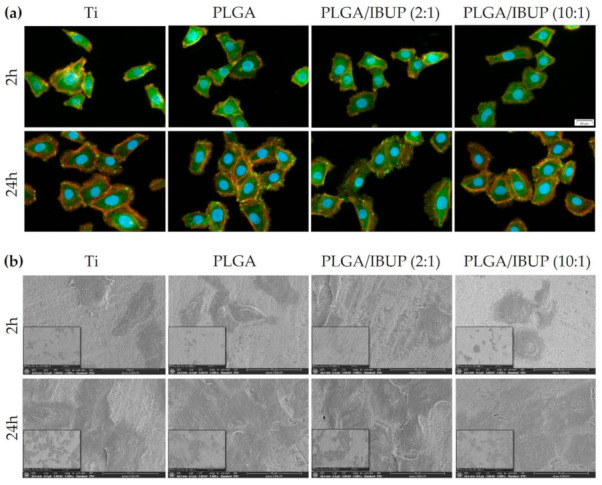
Adhesion and morphology of human keratinocytes on PLGA-based coatings after 2 h and 24 h, evidenced by: (**a**) fluorescence microscopy showing vinculin protein (green), actin filaments (red) and nuclei (blue), scale bar 20 µm; (**b**) SEM investigation, scale bar 40 µm.

To evaluate cell-to-substrate adhesion and cellular morphology of KERTr cells grown on PLGA/IBUP coatings, concomitant visualization of green-labeled vinculin and red-labeled actin was done (Figure 12a). At 2 h post-seeding, comparable cellular densities were observed for all samples, but the preferential polygonal shape of cells and filopodia extension were particularly noticed in the case of metallic substrates modified with PLGA and PLGA/IBUP coatings. At this point, predominant diffuse vinculin signals (green fluorescence) and discreet focal adhesions were observed. All cells exhibited typical polygonal morphology and enhanced focal adhesions (evidenced by punctiform vinculin signals), as distinguished after 24 h of incubation, regardless of the substrate. A weak intracytoplasmic disposal of actin filaments near the cell membrane was noticed after 2 h of incubation, whereas well-defined cortical staining was noticed at 24 h post-seeding. Thus, the developed coatings, including the IBUP-loaded ones, proved the ability to support the adhesion of human keratinocytes.

To get a more complete picture of keratinocyte adhesion and morphology, SEM investigation was performed at the same time points. Thus, the micrographs from Figure 12b reinforced the idea that IBUP-loaded PLGA coatings were beneficial for the attachment and early spreading of keratinocytes, as indicated after 2 h by their surface disposal, preferential polygonal shape and abundant cytoplasmic extensions. By increasing the incubation time to 24 h, KERTr cells in contact with these surfaces were more spread and numerous, suggesting their accommodation to the surface’s features, as well as their potential to proliferate.

**Figure 13:**
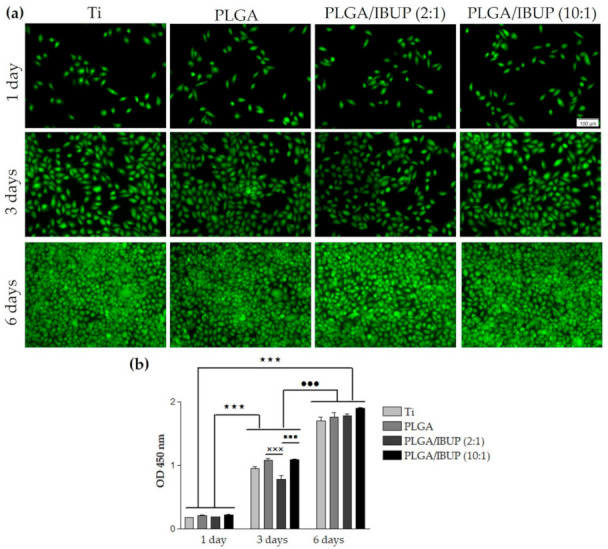
Viability and proliferation of human keratinocytes grown in contact with PLGA-based coatings for 1, 3 and 6 days, evaluated by: (**a**) viable cell fluorescent labeling (Live/Dead assay), scale bar 100 µm; (**b**) keratinocyte proliferative potential (CCK-8 test); ^★★★^ *p* < 0.001 vs. 1-day time point; ^•••^ *p* < 0.001 vs. 3-day time point; ^×××^ *p* < 0.001 vs. PLGA; ^■■■^ *p* < 0.001 vs. PLGA/IBUP (10:1).

We further investigated the potential of developed surfaces to sustain keratinocyte survival and proliferation. The qualitative results of keratinocytes’ viability, evaluated by the Live/Dead assay, are included in Figure 13a. An increase in the number of viable cells was noticed with increasing the incubation time. Even if a slight decrease of viable cells’ density was noticed for bare Ti and PLGA/IBUP (2:1)-coated Ti after 3 days of culture, comparable densities were observed on all specimens after 6 days of incubation. Moreover, there was no evidence of dead cells, which proved the ability of PLGA/IBUP coatings to support viability and promote normal proliferation of KERTr cells. According to Figure 13b, a time-dependent increase in the number of metabolically active human keratinocytes was observed for all samples when the CCK-8 assay was performed. The OD values recorded after 3 days of incubation were reduced for uncoated metallic specimen and Ti modified with PLGA/IBUP (2:1) coating. At this point, the best viability rates were noticed for PLGA and PLGA/IBUP (10:1) materials (*p* < 0.001 vs. PLGA/IBUP (2:1)). However, at 6 days post-seeding, this difference was significantly reduced and collected data showed the high cytocompatibility of the tested materials.

## 4. Conclusions

Uniform composite coatings of Ibuprofen-loaded poly(lactic acid-*co*-glycolic acid), PLGA/IBUP, were obtained by using the dip-coating and drop-casting combined protocol. The gradual polymer degradation and long-term drug release were evaluated by biologically simulated dynamic studies. Complementary compositional and microstructural data evidenced the irreversible degradation of PLGA matrix after longer testing times (20 and 30 days), a process accompanied by a substantial increase in the release of IBUP. The *in vitro* studies on cells known to be the major players in wound healing process (e.g., fibroblasts, keratinocytes and macrophages) demonstrated that the PLGA-based materials equally support the viability and proliferation of fibroblasts and keratinocytes, except for the PLGA/IBUP (2:1) sample after 3 days of keratinocyte culture, which showed a significant decrease as compared to PLGA and PLGA/IBUP (10:1) specimens. However, these differences disappeared at the 6-day time point. In the case of macrophages, the number of metabolically active cells was significantly increased for both PLGA/IBUP coatings when the contact time varied from 1 to 3 days. Additionally, no inflammatory response was induced by the composite materials. Altogether, our study evidenced the potential of PLGA/IBUP coatings to be used for surface modification of medical devices, such as implants and wound dressing.

## Data Availability

The data presented in this study are available on request from the corresponding author.
